# The role of executive functions in the pragmatic skills of children age 4–5

**DOI:** 10.3389/fpsyg.2014.00240

**Published:** 2014-03-20

**Authors:** Bénédicte Blain-Brière, Caroline Bouchard, Nathalie Bigras

**Affiliations:** ^1^Qualité Éducative des Services de Garde et Petite Enfance, Département de Psychologie, Université du Québec à MontréalMontréal, QC, Canada; ^2^Qualité Éducative des Services de Garde et Petite Enfance, Département D'études sur L'enseignement et L'apprentissage, Université LavalVille de Québec, QC, Canada; ^3^Qualité Éducative des Services de Garde et Petite Enfance, Département de Didactique, Université du Québec à MontréalMontréal, QC, Canada

**Keywords:** pragmatic skills, communication, executive functions, vocabulary, visuoconstructive abilities, cognitive development, language acquisition, early childhood

## Abstract

Several studies suggest that pragmatic skills (PS) (i.e., social communication) deficits may be linked to executive dysfunction (i.e., cognitive processes required for the regulation of new and complex behaviors) in patients with frontal brain injuries. If impairment of executive functions (EF) causes PS deficits in otherwise healthy adults, could this mean that EF are necessary for the normal functioning of PS, even more so than cognitive maturation? If so, children with highly developed EF should exhibit higher levels of PS. This study aimed to examine the link between EF and PS among normally developing children. A secondary goal was to compare this relationship to that between intellectual quotient (IQ) and PS in order to determine which predictor explained the most variance. Participants were 70 French-speaking preschool children (3;10–5;7 years old). The PS coding system, an observational tool developed for this study, was used to codify the children's PS during a semi-structured conversation with a research assistant. Five types of EF processes were evaluated: self-control, inhibition, flexibility, working memory and planning. IQ was estimated by tallying the scores on a receptive vocabulary test and a visuoconstructive abilities test. The results of the test of differences between correlation coefficients suggest that EF contributed significantly more than IQ to the PS exhibited by preschoolers during conversation. More specifically, higher inhibition skills were correlated with a decrease in talkativeness and assertiveness. EF also appeared to foster quality of speech by promoting the ability to produce fluid utterances, free of unnecessary repetition or hesitation. Moreover, children with a high working memory capacity were more likely to formulate contingent answers and produce utterances that could be clearly understood by the interlocutor. Overall, these findings help us better understand how EF may assist children in everyday social interactions.

## Introduction

Pragmatic skills (PS) in children refer to the ability to use communication strategies in social interactions (Owens, 2011). These skills contribute to children's psychosocial adjustment and academic achievement (Ervin-Tripp, 1978; McKown, 2007; Coplan and Weeks, 2009; Brinkman et al., 2013). Russell and Grizzle (2008) examined 24 instruments used to assess PS among children and adolescents in order to identify the core domains of PS. They found just over 1000 different items in these instruments, which they grouped into 17 domains and further classified into three sets: (1) Precursors/enablers (e.g., non-verbal communication; discourse attentiveness and empathy; speech characteristics and fluency), (2) Basic exchanges/rounds (e.g., conversational turn taking; topic control and maintenance; requests), (3) Extended literal and non-literal discourse (e.g., negotiations, directions, and instructions; theory of mind; narrative; Gricean principles) (Russell and Grizzle, 2008). Although this classification is helpful, there is still no empirical finding corroborating such a categorization. In fact, Russell and Grizzle (2008) reported that almost none of the authors who constructed the instruments they inventoried had performed factorial analyses. Thus, in order to describe the empirical dimension of PS, specifically among preschoolers, the authors of the present study carried out a systematic literature review and performed a factor analysis (Blain-Brière et al., submitted). They concluded that preschoolers' PS can be divided into five categories: conversational complexity, talkativeness, assertiveness, communicative control and responsiveness.

Studying the development of this five categories of PS in preschooler, a year or two prior to the school commencement, is crucial because it is around this age that children start to play interactively with each other (Smith, 2003). Their ability to manifest PS will shape their early socialization experiences, influence their social acceptance and help them develop their socials skills (Black and Hazen, 1990; McKown, 2007). By preschool age, children have already mastered a wide range of PS (Adams, 2002). By age 1, they know how to request something by pointing to it (Carpenter et al., 1998; Liszkowski et al., 2004). Between the age of 2 and 4, Martinez (1987) shows that children's speech contains more turnabout, namely a utterance that have the dual function of responding to the speaker and restarting the conversation. Pellegrini et al. (1987) note also that children of this age tend to exchange more utterances with their interlocutor, from around 14 utterances per minute at age 2 to about 22 utterances per minute at age 3–4. By about age 3, they can already adapt their speech to an interlocutor (Dunn and Kendrick, 1982). Sachs et al. (1991) showed that at age 3, children have a tendency to ask adults questions regardless if it is an appropriate time to do it, whereas most children by age 5 are able to wait until the adult has finished speaking before querying them. Some abilities, such as understanding figurative speech, are not completely acquired until adolescence or even adulthood (Nippold, 1985; Ervin-Tripp et al., 1990; Spector, 1996).

Developmental studies have thus shown that children are constantly required to manifest PS, and that these skills become increasingly cognitively demanding as they get older. Could cognitive factors therefore play a role in the acquisition of PS? For instance, before children are able to wait their turn to speak, surely they must first acquire the ability to inhibit a response. In order words, inhibition skills, a cognitive process involved in executive functioning, would need to be sufficiently developed before a child could refrain from speaking during his interlocutor's speaking turn. In brief terms, executive functions (EF) are defined as the mechanisms that regulate cognition by modulating the operation of a variety of cognitive processes including inhibition, but also working memory (WM), flexibility and planning (Lehto et al., 2003; Blair et al., 2005). Yet, while the involvement of cognitive processes such as EF in PS seems logical, to date, few authors have investigated this relationship among typically developing children.

In adults, PS deficits (e.g., excessive talkativeness, subject shifting, problems understanding indirect questions) following a prefrontal brain injury are well-documented in the literature (Martin and McDonald, 2003; Douglas, 2010; Dardier et al., 2011). Several studies have found that PS deficits are correlated with executive dysfunction in patients with traumatic brain injury (TBI) (McDonald and Pearce, 1996, 1998; Channon and Watts, 2003; Douglas, 2010). This correlation implies that EF are necessary for the normal functioning of PS. Based on this premise, it seems probable that EF may also contribute to the acquisition of PS in normally developing children (Blain-Brière et al., submitted). Therefore, children with well-developed EF should exhibit better PS than other peers of the same age. Of course, these deductions are theoretical and need to be proven. Yet, there is evidence supporting them. For instance, children with executive dysfunction, caused by a neurodevelopmental disease such as attention deficit hyperactivity disorder (ADHD) (Humphries et al., 1994; Bruce et al., 2006) or autism (Ozonoff, 2001; Norbury et al., 2004; Bishop and Norbury, 2005; Reisinger, 2011; Schuh, 2012), have been found to exhibit PS deficits.

Even among normally developing children, according to Nilsen and Graham (2009) and Schuh (2012), there is proof of a correlation between EF and PS. To evaluate PS, these authors used a similar referential communication experimental protocol that specifically measured how children used speech to signify things in the world. In this task, the participant was typically asked by the examiner to choose an object from an array of objects. The participant had to take into account the context of the situation such as what the examiner could see from his position. For instance, if the examiner could not see the red object from where he was standing, the participant would conclude that the object asked for was not red. In their study among typically developing children aged 3–5 years, Nilsen and Graham (2009) noted that inhibition contributed to the children's ability to consider the perspective of the examiner when choosing the right object. Their interpretation was that inhibition allowed the children to inhibit their own perspective in order to consider the viewpoint of the examiner (Nilsen and Graham, 2009). Schuh (2012) also used a referential communication task to study the influence of WM among typically developing children aged 8–17 years. She demonstrated that children with a higher WM capacity responded more accurately to their partner's request because they were able to take into account information that the latter did not know about the situation. The results of Nilsen and Graham (2009) and Schuh (2012) show that inhibition and WM may increase the ability to interpret the perspective of others. Consequently, children with highly developed EF may be better at grasping the speech of their interlocutor, especially when it is ambiguous, and respond accordingly. This gain in responsiveness during conversation could mean that EF increase PS among children. However, as pointed out by Bishop and Adams (1991), referential communication tasks are not necessarily representative of how children communicate in an unstructured conversational setting. These authors demonstrated, for instance that children who provided excessive and irrelevant information in such a task did not act the same way during open-ended conversation. Hence, the link between PS and EF needs to be demonstrated in a more natural context in order to confirm that EF truly benefit children in conversation. To date, very few studies have examined the relationship between EF and PS through a direct observation measure of PS (Jagot et al., 2001). An observational research design is needed to confirm that children do indeed rely on EF in their everyday social interactions.

Moreover, it is important to note that EF are not the only cognitive processes thought to contribute to PS. In fact, previous research has shown that vocabulary, visuoconstructive abilities and intellectual quotient (IQ) may also be related to PS (McDonald, 2000; Bonifacio et al., 2007; McKown, 2007). Nevertheless, regression analyses have demonstrated that EF may make a unique contribution to the PS of children, even after controlling for vocabulary size and age (Nilsen and Graham, 2009). However, while regression analyses may prove that EF explain a unique part of the variance, they cannot tell which predictor, among vocabulary, visuoconstructive abilities, IQ and EF, has the strongest relationship with PS. On the other hand, a test of differences between correlations would make it possible to determine the relative role played by each predictor and whether these differences are significant. Such an analysis would allow answering the question of (1) whether overall cognitive maturation (e.g., vocabulary, visuoconstructive abilities and IQ) has more or less the same influence on PS as EF or (2) whether each EF process plays a specific role in PS which is significantly different from that played by other cognitive processes.

The above-cited TBI and general population studies have another shortcoming when it comes to demonstrating a link between PS and EF. They usually use a very limited number of measures of PS and/or EF. Douglas (2010), for instance, measured only EF in the verbal domain [verbal fluency (FAS), verbal memory (RAVLT) and speed and capacity of language-processing (SCOLP)]. Nilsen and Graham (2009) and Schuh (2012), for their part, measured only PS related to referential communication. Consequently, these authors could not show exactly how each EF process may contribute to each PS separately.

To further our understanding of the possible role played by EF in the normal acquisition of PS, this study aimed to examine the link between EF and PS among typically developing preschoolers. This study was innovative insofar as it used a direct observational tool to evaluate PS in order to assess how EF might influence the PS of children in their everyday social interactions. Moreover, a test of differences between correlations helped us to understand to what extent the link between EF and PS is different from the relationship between an IQ estimate and PS. This study also adds to previous work in the field by using a wide range of variables to measure PS (14 variables) and EF (self-control, inhibition, WM, flexibility and planning).

## Materials and methods

### Participants

The study sample consisted of 70 French speaking children (34 girls and 36 boys) with an average age of 4 and a half years (55.2 months, *SD* = 4.5 months, 3;10–5;7 years). They were all recruited from a subsidized childcare center in a class designed for children who will enter the school system in a year or two. In order to participate, the children's language had to be developing normally based on the information reported by their childcare provider and the results of a receptive vocabulary task. Eighty children were initially recruited, 10 of whom could not be included in the study, either because of suspected language delays (4 subjects), because the child was absent when the testing took place (3 subjects) or as a result of technical problems during the video recording of the conversation sample (3 subjects). As for the sociodemographic characteristics of the participants, 30.6% lived in a household with an income of less than $30,000, while the household income for 28.7% was $30,000–70,000, for 28.1% was $70,000–100,000, and for 28.1% exceeded the threshold of $100 000. As for the level of education of the participants' mothers, 3.1% of mothers had not completed high school (11 grades in Quebec, Canada), 9.4% had at most a high school education, 12.5% had a vocational school diploma, 26.6% had a college education and 48.4% had a university degree.

### Materials

#### Pragmatic skills

The *Grille d'observation des habiletés pragmatiques des enfants d'âge préscolaire* (Pragmatic Skills Coding System—Preschool Version (PSCS-P) (Blain-Brière et al., submitted) was used to measure PS. This instrument was developed after three years of research by the authors of this article in order to palliate for the lack of validated observational tools for assessing PS among preschoolers. The PSCS-P measures 14 PS parameters during a semi-structured conversation with an examiner. The parameters were developed by selecting variables from 21 utterance coding systems, themselves retrieved from a systematic literature review. To ensure content quality of the parameters selected, independents expert's advices were solicited and factor analysis were performed. Table 1 describes how these variables were codified and their Intraclass correlation coefficient (ICC) measured in the validation process on a sample of 18 participants. It also presents the five scales that they are associated with and their coherence coefficients. This observational protocol, based on a make believe picnic game, was inspired by the Peanut Butter Protocol (Creaghead, 1984). The examiner follows a protocol whereby he invites the child, in a natural way, to express 23 communicative intentions or rules of communication. For example, the examiner may probe the communicative intention “request for action” by asking the child to open a bottle of juice with a cap that cannot be opened by children. The examiners are trained to follow the children's lead if the situation presents itself (e.g., if the children ask a question) in order to promote a natural conversation, while continuing to follow the protocol as they go along. Each of the first 50 utterances produced by the child is coded according to the presence or absence of criteria pertaining to the 14 variables of the PSCS-P, except for the variables “number of words per minute” and “number of utterances per minute,” for which the numbers are tallied. The speech samples of this study were codified by the same person (the principal author) to increase the reliability of this measure. The results are then compiled into an *Excel* file and formulas are used to convert the results into a percentage of success.

**Table 1:** **Description of the pragmatic skills coding system—preschool version**.

**CONVERSATIONAL COMPLEXITY SCALE (α = 0.68)**	
Turnabout	Percentage of utterances that have the dual function of responding to the interlocutor and restarting the conversation by adding information [e.g., “But” (response) “This glass will be mine.” (expansion) (ICC = 0.67^a^)].
Organization of utterances	Percentage of utterances that link more than one piece of information (regarding people, objects, time, location, action, etc.) in a single utterance [e.g., “I'm (subject) gonna eat (action) grapes (object).” (ICC = 0.79)].
Number of new themes	Percentage of utterances that produce new themes (ICC = 0.62).
Abstraction level of themes	Percentage of utterances that introduce themes that are decontextualized in time (e.g., I'm gonna go skiing this winter), place or reality (fictitious/fantasy) (e.g., You you're the mom and I'm the dad (ICC = 0.89).
**TALKATIVENESS SCALE (α = 0.71)**	
Number of words	Number of words per minute (ICC = 1.00).
Number of utterances	Number of utterances per minute (ICC = 1.00).
Number of utterances per speaking turn	Percentage of utterances that express more than one utterance (separated by a delay of more than 2 s) per speaking turn (ICC = 0.93).
**ASSERTIVENESS SCALE (α = 0.66)**	
Initiations	Percentage of utterances that initiate conversation, rather than answering a question (ICC = 0.88).
Requests	Percentage of utterances that formulate requests (ICC = 0.56).
Conversation breakdown repairs	Percentage of utterances that repair conversation breakdowns (e.g., child: “Box.,” research assistant: “What?,” child: “The box.” (ICC = 0.52).
**COMMUNICATIVE CONTROL SCALE (α = 0.38)**	
Fluidity	Percentage of utterances that are free of involuntary and unnecessary repetition or hesitation (e.g., “I want the… the bottle”) (ICC = 0.93).
Non-interruption	Percentage of utterances that do not interrupt the interlocutor (ICC = 0.72).
**RESPONSIVENESS SCALE (α = 0.61)**	
Contingency	Percentage of utterances that adequately respond to a request by the interlocutor (e.g., research assistant: “Will you play with the puzzle?,” child: “OK.”) (ICC = 0.81).
Utterance clarity	Percentage of utterances that express clear and understandable statement (ICC = 0.14^b^).

a ICC, Intraclass correlation coefficient. The speech samples of this study were codified by the same person. However, the principal author and an undergraduate student codify eighteen speech samples separately, during the validation process of the PSCS-P, in order to compute the ICC of each variable.

b This variable's ICC is below the “fair” level of 0.40 suggested by Cicchetti (1994). But when the inter-rater reliability is calculated in terms of percentage of agreement, the rate of this variable still remains relatively high at 91%, even higher than other variables. The lack of variability in this variable seems to have reduced the ICC.

#### Executive functions

Four neuropsychological tests were used to assess self-control, inhibition, flexibility, WM, and planning. Although these tests are not commercialized tools, they are frequently used in research in the absence of tests with better psychometric properties for preschoolers (Monette and Bigras, 2008).

The Prohibited Toy protocol was used to measure *self-control* ability (Rasmussen et al., 2008). This task correlates with other tests involving “hot” inhibition (Monette and Bigras, 2008), which refers to the cognitive process controlling decision-making that entails an emotional or motivational issue (Hongwanishkul et al., 2005; Zelazo and Müller, 2005). In the Prohibited Toy task, the examiner asks the child to turn his back so that they can play a guessing game. After two successful guesses (which animal corresponds to the sound made by a toy animal) the examiner announces to the child that he has to leave for a minute. Before leaving, the examiner asks the child not to look at the object behind him so that they may continue the guessing game upon the examiner's return. No points are awarded if the child looks at the object and one point is attributed if the child does not turn around to look.

The Backwards Digit Span (BDS) was used to assess *working memory* in an auditory-verbal modality (Davis and Pratt, 1995). In Davis and Pratt's protocol (1995), the examiner demonstrates to the child how to repeat a series of two numbers backwards using a puppet. The examiner then notes the longest series of numbers that the child manages to repeat backwards. The child is assigned a score of one if he fails to repeat two digits backwards, a score of two if he can recall two and so on.

The Dimensional Change Card Sort (DCCS) was used to measure *flexibility* (Zelazo, 2006). In this test, the examiner shows the child two target cards, a blue rabbit and a red boat, and asks the child to sort a set of cards, assigning each card either to the “red rabbit” pile or the “blue boat” pile. In the first phase, the child must sort the cards according to the shape of the objects on them. In the second phase, the child must sort the cards according to their colors. In the third phase, the child must alternate between sorting the cards by color and sorting them by shape. The child receives one point if he succeeds in the first phase, two for the second phase and three for the third phase.

The Tower of Hanoï (ToH) was used to measure *planning* and *inhibition* (Welsh et al., 1991). In this test, the child must move three rings of increasing size around on three pegs. The aim is to reach the final position with all the rings in descending order on the peg to the right. This must be done within the least number of moves while observing three rules: (1) not to put a larger ring on top of a smaller one, (2) to move the rings one at a time and (3) not to place the rings anywhere but on the pegs. The examiner explains the rules using an analogy—referring to the rings as a family of squirrels (i.e., smaller = child, medium = mother and larger = father)—and a demonstration. The examiner then makes sure the child understands the rules by asking him to perform the allowed moves. The child is entitled to six trials for each new problem. If he finds the solution within the designated number of moves on the first trial, he is assigned 6 points. One point is subtracted each time the child needs an additional trial to solve the problem within the designated number of moves. If the child fails to solve the problem within the designated number of moves after six trials, the examiner does not administer the following problems. The planning score is computed based on the total number of points, with a maximum score of 36 points (6 points for each of 6 problems). The inhibition score is computed by calculating the number of illegal moves over the total number of trials played (Ahonniska et al., 2000). The term “inhibition” is used here to differentiate it from the self-control measure evaluated by the Prohibited Toy protocol. The inhibition score on the ToH can be considered a cool type of inhibition because, as opposed to the Prohibited Toy protocol, the goal of the task is more cognitive and has no emotional underpinning (Hongwanishkul et al., 2005; Zelazo and Müller, 2005).

A principal component analysis (PCA) was performed on the EF measures to ensure that it was statistically possible to create a composite score with these measures. The flexibility score, however, was removed from the composite score because of its lack of interindividual variability (see Table 2) and the absence of any significant correlation with the other EF measures (*r* = 0.06 to 0.22, *p* > 0.05). The PCA resulted in a one-factor solution, explaining 56.63% of the variance in the four remaining EF scores. Consequently, the composite score was computed by tallying the scores of each measures in standardized score.

**Table 2:** **Descriptive statistics for the executive function (EF), intellectual quotient (IQ), and pragmatics skills (PS) measures**.

**Constructs**	**Measures**	**Range**	**Min-max**	**Scores distribution (%)**			**Means**	***SD***
				**Low**	**Medium**	**High**		
**PS**								
Conversational complexity	PSCS-P	0–4	0.08–1.47	33	34	33	0.77	0.34
Talkativeness	PSCS-P	0–3	0.34–1.63	33	34	33	0.91	0.32
Assertiveness	PSCS-P	0–3	0.45–2.56	33	34	33	1.51	0.52
Communicative control	PSCS-P	0–2	1.73–2.00	33	34	33	1.88	0.07
Responsiveness	PSCS-P	0–2	1.56–2.00	31	38	30	1.84	0.09
**EF**								
Self-control	Forbidden toy	0–1	0–1	66	–	34	0.61	0.49
Inhibition	Towers of hanoï^a^	0–1	0.14–0.92	31	36	30	0.52	0.23
Working memory	Backward digit span	1–5	1–4	44	31	24	1.82	0.83
Flexibility	DCCS	1–3	1–3	17	71	11	1.94	0.54
Planning	Towers of hanoï^b^	0–36	0–32	33	34	33	15.29	8.37
**IQ ESTIMATE**								
Vocabulary	PPVT-R (French version)	0–175	23–93	33	34	33	60.59	18.24
Visuoconstructive abilities	Block design (WPPSI-III)	0–40	18–32	37	23	40	24.08	3.03

Raw scores are presented.

a Number of illegal moves over the total number of trials played.

b Problem resolution scores.

#### Estimated intellectual quotient

The Peabody Picture Vocabulary Test—Revised (PPVT-R, French version) (Dunn et al., 1993) and the Block Design from the Wechsler Preschool and Primary Scale of Intelligence, 3rd edition (WPPSI-III) (Wechsler, 2002) were chosen to represent verbal (Fagan et al., 2007) and non-verbal IQ (Sattler, 2008). The PPVT-R evaluates *receptive vocabulary*. In this task, the child is presented with a set of four pictures. The examiner asks the child to point to the picture that corresponds to the word he says. The Block Design from the WPPSI-III was used to assess *visuoconstructive abilities*. In this test, the child is asked to reproduce several two-dimensional models with blocks, as fast as he can. The raw results of these tests were used for the purposes of analysis to facilitate comparison with the EF tests, for which normative data were not available.

A second PCA was performed on the measures used to estimate IQ, namely, vocabulary and visuoconstructive abilities, with the objective of creating another composite score. A one-factor solution emerged explaining 61.52% of the variance. Thus, the PCA supported the aggregation of the results for vocabulary and visuoconstructive abilities into an IQ composite score. Again, results were computed by adding the scores for each of the measures in standardized score.

### Procedure

Participants were recruited in the fall of 2008. The participating children were recruited through five publicly funded childcare centers in the Montreal region. Parental consent for the participants' participation in the research project was given following a request by email and phone. The instruments were administered by three psychology students who had received 15 h of training on the administration of the instruments. Each child was individually tested at his childcare center during two 45-min periods. The examiners administered the PPVT-R and the observational protocol of the PSCS-P on the first day of testing. On the second day, they administered, in the following order, the Block Design subtest (WPPSI-III), the DCCS, the Prohibited Toy protocol, the BDS and the ToH. The childcare provider and the participating children received a book to thank them for their participation.

## Results

Table 2 presents the descriptive results for all the measures: (1) PS, evaluated using the five scales of the PSCS-P (conversational complexity, talkativeness, assertiveness, communicative control and responsiveness), (2) EF, assessed through measures of self-control, inhibition, WM, flexibility and planning and (3) IQ, estimated based on measures of receptive vocabulary and visuoconstructive abilities. In order to determine the distribution of participants across these measures, their scores were divided into three categories: low, medium and high. It should be noted that the children's PS, EF, and IQ scores were generally fairly well-distributed across these different categories. However, 71% of the children were assigned a medium flexibility score on the DCCS, which means that this measure showed very low interindividual variability.

Prior to all inferential statistics, transformations were made to the data to reduce the inconvenience caused by missing data when administering the EF tests. These missing data (4.5%) were replaced by an algorithm of Expectation Maximization (EM) by calculating the expected scores based on the results of the other EF scores. This method was chosen because the missing data were randomly distributed across the various measures [MCAR Chi2 (8) = 14.35, *p* > 0.05] (Ervin-Tripp, 1978). In addition, one subject had a multivariate extreme value, detected by calculating the Mahalanobis D2. This subject's results on the ToH were very abnormal and thus were replaced by an EM algorithm using the results of the other EF tests. Moreover, some of the variables of the PSCS-P were not normally distributed. Logarithmic transformations were performed to normalize the “breakdown repairs,” “non-interruption,” “contingency,” and “utterance clarity” variables. The “abstraction level of themes” variable was dichotomized based on the presence or absence of at least one decontextualized theme during the exchange.

Before addressing the main objective of this study, Pearson correlations performed in order to present the link between the sociodemographics characteristics, namely, age, gender, household income and education of the mother, and our measurements. These correlations, presented in Table 3, show that mother education has the strongest relation with children performance on the measure of PS, EF, and IQ (ranging from *r* = −0.10, *p* > 0.05 to *r* = 0.32, *p* < 0.01). Both age and income correlate significantly with vocabulary (respectively *r* = 0.26, *p* < 0.05 and *r* = 0.36, *p* < 0.01) and planning (respectively *r* = 0.33, *p* < 0.01 and *r* = 0.30, *p* < 0.05) for instance. On the other hand, gender is only significantly associated with talkativeness (*r* = 0.27, *p* < 0.05), indicating that boys are more talkative than girls.

**Table 3:** **Pearson correlations between sociodemographic characteristics and executive functions (EF), intellectual quotient (IQ), and pragmatics skills (PS) measures**.

	**Age (month)**	**Gender**	**Income**	**Mother's education**
**PS**				
Complexity	−0.07	0.17	−0.05	0.31^*^
Talkativeness	−0.14	0.27^*^	−0.18	0.15
Assertiveness	−0.22	0.05	0.04	0.28^*^
Communicative control	0.08	0.07	−0.02	−0.10
Responsiveness	0.17	0.18	−0.19	−0.07
**EF**				
Self-control	0.02	−0.15	−0.02	−0.09
Inhibition	0.23	−0.10	0.25^*^	0.14
Working memory	0.12	0.06	0.05	0.27^*^
Flexibility	0.32^**^	0.10	0.24	0.32^**^
Planning	0.33^**^	−0.02	0.30^*^	0.30^*^
**IQ ESTIMATED**				
Vocabulary	0.26^*^	0.15	0.36^**^	0.19
Visuoconstructive	0.15	0.22	0.02	0.28^*^

* p < 0.05,

** p < 0.01.

As for the inferential statistics, Table 4 presents the Pearson correlations performed to determine what role self-control, inhibition, flexibility, WM, planning and the EF composite score (sum of all EF measures except flexibility) played in the children's PS. In order to determine whether the contribution of EF to PS was significantly different from that of IQ to PS, differences among the correlation coefficients were tested using the Fisher *z* transformation formula proposed by Meng et al. (1992). On the whole, these analyses showed that EF correlated with PS differently than IQ for 2 of the 5 scales in the PSCS-P and 3 of the 14 associated variables (see Table 4).

**Table 4:** **Pearson correlations between pragmatic skills (PS) and executive functions (EF) and between PS and intellectual quotient (IQ); and results of the test of differences between the correlation coefficients for the two relationships**.

**PS**	**EF**					**IQ**		**EF**^a^****	**IQ**^b^****	**rPSxEF ≠ rPSxIQ**^c^****	
**Scales variables**	**Self-control**	**Inhibition**	**WM**	**Flexibility**	**Planning**	**Vocabulary**	**VC**			***Z***	**(*p*)**
Conver. complexity	−0.20	−0.09	0.03	0.20	−0.10	0.09	0.18	−0.12	0.18	2.10	0.04^*^
Turnabout	−0.12	−0.07	0.03	0.20	−0.10	0.05	0.16	−0.09	0.14	1.61	0.11
Organization of utterances	−0.18	−0.15	0.06	0.10	−0.05	0.17	0.20	−0.11	0.23^t^	2.38	0.02^*^
Number of new themes	−0.20	−0.07	0.01	0.16	−0.06	0.12	0.10	−0.11	0.14	1.75	0.08
Abstraction level of themes	−0.09	0.03	0.04	0.14	−0.11	−0.08	0.12	−0.04	0.03	0.63	0.53
Talkativeness	−0.19	−0.28^*^	−0.09	0.04	−0.15	−0.03	0.10	−0.24^*^	0.05	2.04	0.04^*^
Number of words	−0.24^t^	−0.18	−0.05	0.07	−0.15	0.05	0.03	−0.21	0.05	1.83	0.07
Number of utterances	−0.14	−0.09	−0.02	−0.04	−0.04	−0.17	0.02	−0.10	−0.09	0.07	0.94
Utterances per speaking turn	−0.10	−0.40^**^	−0.15	0.07	−0.18	0.05	0.18	−0.28^*^	0.15	3.02	0.002^**^
Assertiveness	−0.23^t^	−0.20	−0.17	0.14	−0.08	−0.01	0.02	−0.23^t^	0.01	1.69	0.09
Initiations	−0.19	−0.20	−0.12	0.16	−0.15	0.14	0.01	−0.22	0.09	2.17	0.03^*^
Requests	−0.12	−0.22	−0.25^*^	0.06	−0.04	−0.12	−0.02	−0.21	−0.09	0.85	0.39
Breakdown repairs	−0.24^t^	−0.05	−0.03	0.11	−0.01	−0.05	0.05	−0.11	−01	0.70	0.48
Communicative control	0.21	0.15	0.13	−0.01	0.26^*^	0.02	0.01	0.25^*^	0.02	1.63	0.10
Fluidity	0.30^*^	0.30^*^	0.25^*^	−0.06	0.31^**^	0.17	0.08	0.38^**^	0.15	1.69	0.09
Non-interruption	0.02	−0.07	−0.05	0.05	0.09	−0.14	−0.06	−0.01	0.13	0.98	0.33
Responsiveness	−0.04	0.00	0.29^*^	0.04	0.00	−0.06	0.09	0.08	0.02	0.42	0.67
Contingency	−0.06	0.03	0.25^*^	0.05	−0.04	−0.06	−0.01	0.07	−04	0.77	0.44
Utterance clarity	−0.02	−0.03	0.26^*^	0.01	0.03	−0.05	0.17	0.08	0.08	0	1.00

VC, visuoconstructive abilities; Conver. Complexity, conversational complexity.

a Flexibility was not included in the EF composite score.

b IQ was estimated using measures of receptive vocabulary and visuoconstructive abilities.

c Probability that the correlation between EF and PS is significantly different (p < 0.05) from that between IQ and PS using the Meng et al. (1992) method.

t Marginally significant at p < 0.06,

* p < 0.05, and

** p < 0.01.

These correlation results are presented in more detail according to each of the five categories of PS: conversational complexity, talkativeness, assertiveness, communicative control and responsiveness. With respect to conversational complexity, no relationship between EF and PS was strong enough to reach the significance threshold. However, the conversational complexity scale (*z* = 2.10, *p* < 0.05) and its variable related to the level of organization of the information in the utterances (*z* = 2.38, *p* < 0.05) correlated significantly differently with EF than with IQ. Specifically, the EF correlation showed a negative tendency with regard to these PS, whereas the IQ correlation showed a positive tendency. Although the EF and IQ correlations with these PS were not significant, the fact that they went in opposite directions resulted in a significant difference.

Regarding talkativeness, both the EF composite score and inhibition were associated with a decrease in the talkativeness scale (*r* = −0.24 and −0.28, *p* < 0.05). They were also related to a decrease in the variable of this scale measuring the number of utterances per speaking turn (*r* = −0.28, *p* < 0.05 and *r* = −0.40. *p* < 0.01). Moreover, self-control was related to a reduction in the number of words per minute, at a marginally significant level (*r* = −0.24, *p* < 0.06). For talkativeness (*z* = 2.04, *p* < 0.05) and number of utterances per speaking turn only (*z* = 3.02, *p* < 0.01), the strength of the EF correlation coefficients differed significantly from the strength of the IQ correlation coefficients. In fact, IQ was related to an increase in these three PS, but did not make a significant contribution to them.

Furthermore, assertiveness yielded a similar correlation pattern to talkativeness and conversational complexity. Again, EF showed a more negative tendency, whereas IQ showed a more positive correlation with PS in general. WM was correlated significantly with a reduction in the number of requests (*r* = −0.25, *p* < 0.05). Three other marginally significant relationships involving EF were also found, all of them being negative. One of these relationships showed that the EF composite score was correlated with the assertiveness scale (*r* = −0.23, *p* < 0.06). The other two showed that self-control was related to a reduction in the number of communication breakdown repairs (*r* = −0.24, *p* < 0.06) and a decrease in the assertiveness scale in general (*r* = −0.23, *p* < 0.06). Although none of the predictors were significantly correlated with the capacity to initiate conversation, the correlation coefficients for EF (*r* = −0.22, *p* > 0.05) and IQ (*r* = 0.09, *p* > 0.05) were significantly different from one another (*z* = 2.17, *p* < 0.05). Once again, the difference in the direction of the correlation, EF being negative and IQ being positive, helped produce a significantly different correlation coefficient between the two predictors.

This difference in the direction of the predictor's relationship with PS was not observed for the communicative control and responsiveness scales. In fact, both EF and IQ tended to correlate positively with these PS and no correlation coefficient differed significantly. As regards communicative control, the most striking result was certainly that all of the measures included in the EF composite score were correlated with utterance fluidity (*r* = 0.25, *p* < 0.05 to *r* = 0.31, *p* < 0.01).

As for responsiveness, WM was positively correlated with the responsiveness scale (*r* = 0.29, *p* < 0.05) and its two variables, namely, contingency (*r* = 0.25, *p* < 0.05) and utterance clarity (*r* = 0.26, *p* < 0.05). No other predictor was correlated with the responsiveness scale or its variables.

It should be noted that no significant correlations were found between PS and the IQ composite score, or the variables on which it was based, namely, vocabulary and visuoconstructive abilities. Nevertheless, there was a marginally significant relationship between IQ and the level of organization of the information in the utterances, a variable associated with the conversational complexity scale.

Given that more than one EF process correlated with utterance fluidity, a standard multiple regression analysis was performed between utterance fluidity (VD) and self-control, inhibition, WM and planning (VI) to calculate the total percentage of explained variance. The four VIs explained 15.2% of the variance associated with utterance fluidity [*F*_(4, 69)_ = 2.91, *p* < 0.05]. Table 5 presents the beta coefficients for each individual predictor, none of which made a unique contribution to utterance fluidity. In others words, if the other predictors were held constant, none of these EF processes would contribute significantly to utterance fluidity.

**Table 5:** **Summary of standard multiple regression analysis for the executive functions processes predicting utterance fluidity**.

	***B***	***SE B***	**β**	***p***
Constant	0.87	0.02	–	
Self-control	0.03	0.02	0.20	0.15
Inhibition	0.02	0.04	0.06	0.72
Working memory	0.04	0.04	0.15	0.23
Planning	0.03	0.04	0.13	0.43

Additionally, partial Pearson correlations were performed in order to control for the sociodemographics characteristics in the relationship between PS, EF, and IQ. Table 6 presents Pearson correlations without others variables accounted for, and the partial Pearson correlations controlling, respectively, for age, gender, income, and education of the mother. Overall, results show little change in the significant level of the correlation after the control of the sociodemographics characteristics (those changes are highlighted in Table 6). In few instance the correlations became non-significants. Those instances involve for the most part the correlations implicating WM when controlling for age, gender, or income. It important to note that age, gender, and income did not make a significant contribution to WM (see Table 3) and therefore, the control of those variables seems to have introduced noise in the model. In others cases, mostly relating to the control of the education of the mother, the correlation significance level was raise.

**Table 6:**
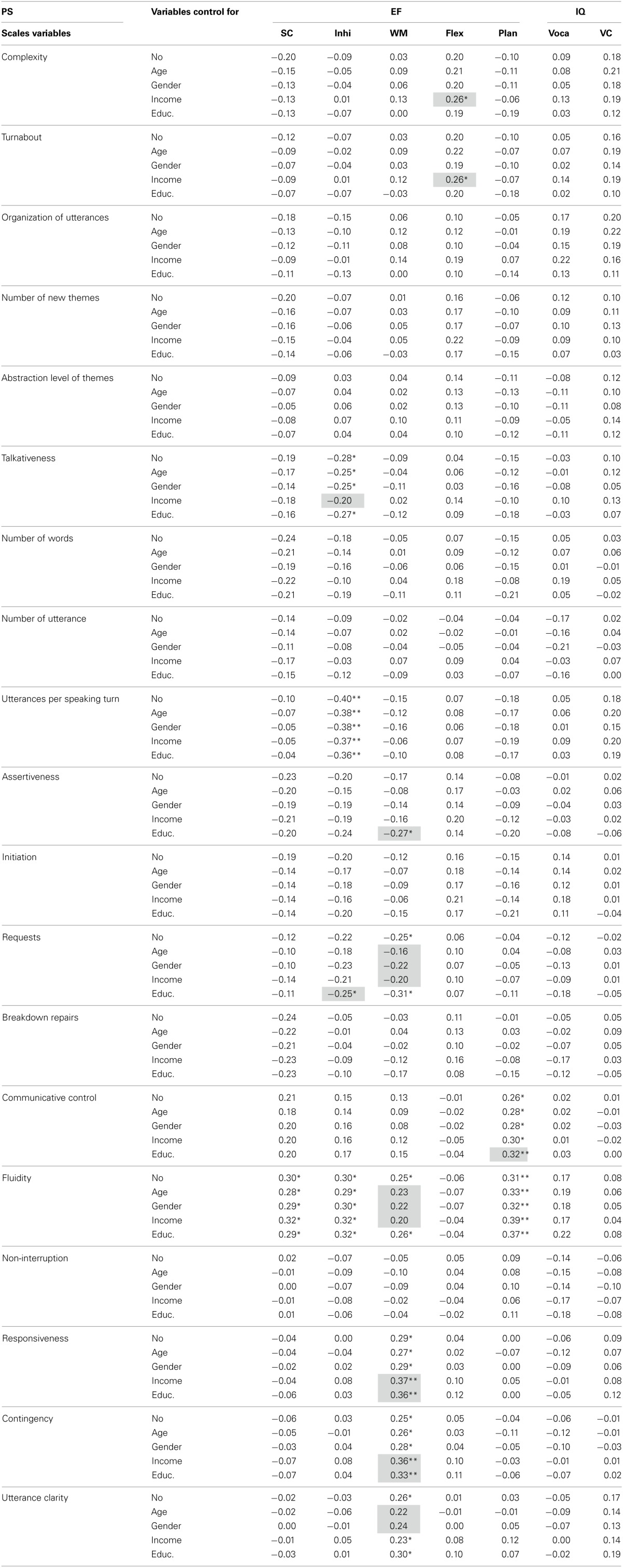
**Partial Pearson correlations between pragmatics skills (PS) and executive functions (EF) and intellectual quotient (IQ) controlling for age, gender, income, and education of the mother**.

SC, sefl-control; Inhi, inhibition; WM, working memory; Flex, flexibility; Plan planning; Voca, vocabulary; VC, vioconstructive abilities; Educ., education of the mother. The shaded cells indicate a change in the level of significance in the correlations between PS, EF, or IQ after controlling the sociodemographic characteristics.

^*^p < 0.05, ^**^p < 0.01.

## Discussion

The objective of this study was to further our understanding of the role of EF in the PS displayed by normally developing children while conversing with an adult. EF are generally defined as processes involved in new and complex tasks (Lezak et al., 2012), as are often social interaction where children most deploy their PS. For example, children between 5 and 7 years are likely to emit more than ten verbal and non-verbal behaviors to integrate a group of peers during play time (Dodge et al., 1986). To express these behaviors in a socially appropriate way, it seems logical to believe that EF like the capacity to anticipate the reactions of other, to plan behavior ahead, to adjust it along the way and to inhibit inappropriate behavior are involved.

Our results show, for instance that higher inhibitory control is associated with a decrease in talkativeness. This result could, at first glance, appear to be counter intuitive since EF should logically assist children with their PS rather than being detrimental to them. Nevertheless, our data are consistent with findings showing an excessive increase in talkativeness among individuals who likely have an inhibition deficit, such as children with ADHD (Landau and Milich, 1988; Humphries et al., 1994; Bruce et al., 2006) and patients with frontal lesions (Bernicot and Dardier, 2001). Arbuckle et al. (2000) revealed a more direct link between low inhibitory control and the tendency to provide more redundant information and be more talkative (marginally significant) among older adults (63–95 years) in a referential communication task. These authors alleged that poorer inhibitory skills could be associated with the intrusion of unnecessary information. It is a well-known finding that inhibitory control is needed to refrain from committing an intrusion error, for example, by retrieving the wrong word in a memory task (Levy and Anderson, 2002). In our study, children who made a greater number of illegal moves in the ToH task had a tendency to produce more than one utterance per speaking turn. This result was one of the more substantial effects found, as approximately 16% of the explained variance in the number of utterances per speaking turn could be accounted for by inhibitory control. It may be that children with higher inhibition skills are better at refraining from speaking more than is necessary, in this case, producing more than one utterance before their interlocutor started to speak again. In this sense, the rules of communication (e.g., respecting speaking turns) may act like the rules of a neuropsychological test such as the ToH. Between the age 2 and 4, Pellegrini et al. demonstrated that children tend to violate less frequently Gricean principles stipulating, for instance that an intervention should bring enough information, but not more than necessary (Grice, 1975). Thus, the decreased in talkativeness might perhaps indicate an increasing in the ability to follow this principal.

Another result was even more unexpected. Indeed, our data show marginally significant correlations between higher self-control (“hot” inhibition) and a decrease in the assertiveness scale and a reduction in the number of communication breakdown repairs. This was also a counter intuitive result since our measure of assertiveness was constructed as a positive concept. Notwithstanding, this result could be consistent with data showing that a lack of inhibition may lead to aggressive behavior (Raaijmakers et al., 2008), which could be viewed as a rare and high amplitude subclass of assertive behavior (Patterson et al., 1967; Ostrov et al., 2006). Of course, correcting the interlocutor's miscomprehension does not correspond to an aggressive behavior because it does not harm this person in any way.

Yet, it is important to recall that the participants in our study were asked to interact with a research assistant with whom they were unfamiliar. Typically, children are much more reserved with an adult with whom they are not acquainted, which may tend to reduce their overall level of assertiveness. In fact, Bishop et al. (1994) showed that, compared to children with Semantic-Pragmatic Disorder, normally developing children had a slightly greater tendency (although not significant, *p* = 0.09) to initiate conversation with a familiar adult than with an unfamiliar one. This means that a low degree of assertiveness with an unfamiliar adult could be a sign of better PS, meaning that the child is able to adapt to the context of the situation. In our observational protocol, the examiner asked the child what color of grapes he wanted and then gave him the other color on purpose. This procedure was used to see whether the child would repair the communication breakdown. As said previously, children with better self-control tended to refrain from correcting the research assistant. If we consider the perspective of a 4 year-old child meeting an unfamiliar adult, it is easy to see why the child might be intimidated by the adult and refrain from correcting him. On the other hand, a child with low self-control may be more inclined to act the same way in any situation, and thus be more likely to correct the research assistant as he would do with a friend. Consequently, self-control may help children refrain from overly asserting themselves when the situation precludes it. Also, we did not take into account the manner used to correct the adult. Future research is needed to evaluate the relationship between the quality of assertiveness and EF, as opposed to the quantity measure used in our study.

Moreover, the above-mentioned negative correlations between inhibition and talkativeness and between self-control and assertiveness lead us to question the linear design of these scales, which presume that a higher score is always better. It may instead be that the ideal level of talkativeness and assertiveness is moderate, neither too high nor too low. Thus, the child should try to adapt to his interlocutor by speaking about the same amount as the latter and acting more thoughtfully, and the child's inhibition level may help him to achieve this.

Furthermore, one of the most impressive findings of this study is the involvement of all EF measures (except flexibility^1^) in the production of more fluid utterances. These results corroborate those of Engelhardt et al. (2013) showing that inhibition was linked to a decrease in dysfluencies among adolescents and adults in a sentence production task. According to their study and previous others (Berg and Schade, 1992; Dell et al., 1997; Engelhardt et al., 2010), inhibition may help reduce the risk of articulating the wrong word by inhibiting the competing phrasing. Our data confirm the entanglement of both “hot” (i.e., emotional) and “cool” (i.e., cognitive) types of inhibition in utterance fluidity in a more natural setting. They also suggest the involvement of WM and planning. On the other hand, vocabulary and visuoconstructive abilities did contribute significantly to the articulation of fluid utterances. Yet, their correlations were not significantly different from those between EF and utterance fluidity, meaning that their role is not much different.

Moreover, the children in our study with a high WM capacity were more likely to formulate contingent answers and produce utterances that could be clearly understood by the interlocutor. They also had a tendency to make fewer requests. The WM or its verbal counterpart, phonological short-term memory, has long been suspected to be involved in language comprehension and production in general (Bock, 1982; Gathercole and Baddeley, 1990; Just and Carpenter, 1992). It has been proposed that the primary function of phonological short-term memory may be to support the long-term learning of the phonological structure of language (Baddeley et al., 1998; Gathercole et al., 2005). There is evidence of this theory in others studies involving speech samples from young children. Indeed, phonological short-term memory in 3–4 year old children has been linked to their ability to formulate more complex utterances in terms of the structural aspects of language, such as the number of words, syntax and vocabulary variety (Adams and Gathercole, 1995; Gathercole, 2000). Although fewer studies have focused on the social aspect of language, there is nevertheless data demonstrating the involvement of WM in PS. For instance, WM has been associated with the interpretation of irony among normally developing children aged 5–9 years (Filippova and Astington, 2008). This increase in language comprehension, and even social understanding, may help children better grasp the situation at hand and consequently respond in a more socially appropriate way.

The negative correlation between WM and the number of requests was more surprising, since requests are sometimes viewed as a more complex communicative intention (Favre and Maeder, 2002). According to our qualitative observations while coding the children's utterances, many of their requests had to do with comprehension (e.g., “What?”). As previously stated, WM is essential for language comprehension. In this sense, children with lower WM may have had more difficulty understanding the interlocutor's speech than other children and may therefore have asked more questions to improve their comprehension. Future studies are needed to confirm this interpretation, especially since we did not measure which types of requests WM was related to.

In sum, EF appear to help preschool children better filter speech, control their level of assertiveness, refrain from articulating utterances incorrectly and respond in a socially appropriate way. Verbal and non-verbal cognitive abilities appear to offer a small, but positive contribution to PS. The effect of EF, on the other hand, appears to be greater than and not always in the same direction as that of IQ. Therefore, EF processes appear to affect PS in a unique and specific way, separately from the more global affect driven by cognitive maturation. Overall, our results suggest that EF play a more important role than IQ in the PS exhibited by children in a semi-structured conversational setting. Indeed, receptive vocabulary and visuoconstructive skills, which were combined to estimate IQ, did not make a significant contribution to any PS. Perhaps the new and unpredictable characteristics of live social interaction are more likely to involve EF.

### Limitations

It is important to note that our results indicate that the influence of EF and IQ on pragmatic skills is generally limited. This means that a large part of the variance can still be accounted for by other factors such as the child's temperament (Coplan and Weeks, 2009) or socialization experiences (Bruner, 2002). Yet, some studies have found a much larger effect size between EF and PS. Douglas (2010), for instance, reported that, among adults with severe TBI, as high as 37% of PS variation (evaluated using the La Trobe Communication Questionnaire) could be explained by executive functioning. In comparison, the strongest relationships found in our study were approximately 15% of explained variance, less than half of the effect size found by Douglas (2010). It could be argued that our sample was composed of a relatively homogeneous group of children (all aged between 3;10 and 5;7 years, typically developing, attending childcare in the same area and mostly raised by educated mothers). This homogeneity may have reduced the variability in our measures and thus the strength of the correlations we were able to obtain.

The lack of interindividual variability seems to have predominantly affected the ability to measure the relationship between flexibility, as measured using the DCCS, and the other variables. Indeed, the flexibility score could not discriminate between the children in our sample (71% of the children had the same score) and did not correlate significantly with the other variables in our study. It would therefore be pertinent in the future to use more sensitive measures of flexibility to differentiate between different levels of cognitive flexibility among 4–5 year old children. Monette and Bigras (2008) have suggested that Hughes' (1998) set-shifting task and the Trail Making Test for preschoolers (Espy and Cwik, 2004) could serve as alternatives to the DCCS, particularly for typically developing children in this age group. Future research could alternatively use a more widespread aged group to increase the interindividual variability of this measure.

It is also necessary to recall the exploratory nature of this study. A large number of statistical analyzes were performed, which has the effect of increasing the probability of a family-wise error rate. Further studies are needed to replicate these results, especially since this is the first study to have used the PSCS-P to examine the link between PS and EF. In addition, the EF tests used in this study did not come from commercialized tools since few such tools are available for preschoolers (Monette and Bigras, 2008). More research should be conducted to develop and validate EF measures for children in this age group. We should also specify that our results came from a single measurement time, thus making it impossible to study the effect of EF on the development of pragmatics. A longitudinal study using multiple time points would make it possible to examine the cognitive factors underlying the acquisition of pragmatics.

## Conclusion

To conclude, research into the cognitive factors that contribute to the acquisition of pragmatics among children is in the beginning stages. Further research involving normally developing children is needed in order to better understand how children acquire pragmatic skills, an ability that is essential to their social development and academic achievement (Ervin-Tripp, 1978; Black and Hazen, 1990; Lemelin and Boivin, 2007; McKown, 2007; Coplan and Weeks, 2009; Brinkman et al., 2013).

### Conflict of interest statement

The authors declare that the research was conducted in the absence of any commercial or financial relationships that could be construed as a potential conflict of interest.

## Footnotes

^1^This absence of a significant relationship may be caused by a lack of interindividual variability, as previously stated.
